# Associations Between Fall Risk and Lower Limb Joint Range of Motion During Sit-to-Stand Among Community-Dwelling Older Adults

**DOI:** 10.53520/rhm2026.104188

**Published:** 2026-01-30

**Authors:** Kworweinski Lafontant, Carla Stokes Leinbach, Nicolle Armas, Patria Marcano Maldonado, Jethro Raphael M. Suarez, Chitra Banarjee, Janet Lopez, Jeffrey R. Stout, David H. Fukuda, Ladda Thiamwong

**Affiliations:** 1 Institute of Exercise Physiology and Rehabilitation Science, College of Health Professions and Sciences, University of Central Florida, Orlando, FL, USA; 2 Elizabeth Morse Genius Foundation Healthy Aging Research Lab, College of Nursing, University of Central Florida, Orlando, FL, USA; 3 Department of Mechanical and Aerospace Engineering, College of Engineering and Computer Science, University of Central Florida, Orlando, FL, USA; 4 College of Medicine, University of Central Florida, Orlando, FL, USA; 5 Disability Aging & Technology Cluster, University of Central Florida, Orlando, FL, USA

**Keywords:** Azure, Kinect, Assessment, Screening, Kinematics

## Abstract

**Introduction:**

Despite the sit-to-stand transition being an acute period of increased fall risk among older adults, it is currently unclear how lower-limb joint range-of-motion (ROM) during this task relates to fall risk.

**Methods:**

We examined 45 community-dwelling older adults (female = 32 (71.1%), age = 76.5±6.1years, BMI = 26.4±4.0kg/m^2^) within this cross-sectional study, assessing lower-limb mobility during 10 seconds of sit-to-stand repetitions using a single camera marker-less system. Hip and knee flexion, hip adduction, and ankle dorsiflexion were assessed for the right and left limbs, as well as asymmetry indices between sides. Fall risk was assessed using the STEADI checklist, Short Physical Performance Battery (SPPB), lower-body muscular power, postural sway, the Short Fall Efficacy Scale-International (FES-I), and 4-meter walking gait speed. Spearman’s rho (ϱ) and Kruskal Wallis H-test were used due to nonnormal data distributions.

**Results:**

High fall risk participants had significantly lower muscular power (2.8±0.6W/kg, p = 0.004, ε^2^ = 0.187) and higher knee flexion asymmetry (4.5±2.5°; p = 0.021, ε^2^ = 0.121) than low fall risk participants (3.6±1.0W/kg; 2.9±2.4°). All correlations between joint movements and fall risk assessments were relatively small/weak (ϱ ≤ |0.38|). Hip flexion asymmetries were significantly correlated with SPPB performance (ϱ = −0.33, p = 0.03) and Short FES-I scores (i.e., fear of falling; ϱ = 0.35, p = 0.02).

**Conclusions:**

Lower-limb ROM during the sit-to-stand transition may not be a strong indicator of fall risk on its own but may provide clinically relevant insight when paired with a validated fall risk assessment.

## Introduction

Falls are a leading cause of injurious death among older adults within the United States, costing approximately $50 billion annually in medical expenses.^1^ Clinicians aim to routinely screen for fall risk factors among older adults as a primary prevention strategy. Fall risk factors include postural sway, physical function, body composition, and mobility. Regarding mobility, however, there is little-to-no consensus on screening methods.

Mobility refers to the ability to move a joint through an expected range-of-motion (ROM),^2^ yet in previous research regarding older adults and fall risk, mobility has largely been defined as the ability for a person to move freely without restrictions or impediments.^3,4^ Although some conceptual overlap does exist between those two definitions, they differ in assessment methods. This is likely why a 2019 systematic review identified 31 different mobility assessments, which largely included gait speed tests, lower-body physical function tests such as the Short Physical Performance Battery (SPPB) and sit-to-stand (STS), and balance tests.^5^ While these tests have a demonstrated relationship with fall risk in past research, they may not be a valid representation of joint mobility and its relationship with fall risk. Joint ROM metrics may inform clinicians about physical fall risk due to disadvantageous movement patterns, such as excessive knee valgus manifesting through increased hip adduction, which has been suggested as an indicator of reduced gluteus medius strength.^6^ Likewise, kinematics at other joints such as the ankle and knee may provide non-invasive insight into fall risk due to neuromuscular attributes that would require additional time and equipment to reliably assess.^7^ However, research directly investigating the relationship between lower-limb joint ROM and fall risk is scant, signifying a meaningful gap in clinical care for older adults.

Within clinical settings, methods used to screen for fall risk must be practical and timely, with a recommended duration of no more than three minutes for a single test.^8,9^ Historically, ROM has been assessed manually using a goniometer during passive movement, which may be of challenge in respect to the time it takes to achieve a reliable measure and difficulties assessing ROM during dynamic tasks. Recent technological advances have helped remedy that issue, with marker-less camera systems demonstrating an ability to reliably assess ROM and kinematics during dynamic movements.^10,11^ Yet, research utilizing this technology for fall risk screenings is limited. A 2014 study by Colagiorgio et al. analyzed lower-limb joint ROM using a single camera Microsoft Kinect system during static (e.g., center-of-pressure postural sway; PS) and dynamic (e.g., STS) tasks and appraised fall risk based on the ROM assessments as well as a modified fall risk assessment conducted by a trained observer.^10^ Their results indicated an 82% accuracy and 83% sensitivity towards fall risk classification (high vs. low risk) using the ROM assessment when compared to the trained observer.^10^ A similar study by Kargar et al. utilized a single camera Microsoft Kinect system to assess lower-limb joint ROM during a Get-Up-and-Go test and a K-cluster statistical design to classify fall risk, reporting an approximate 67% accuracy of the ROM assessment’s fall risk classification.^11^

While these results demonstrate the potential clinical application of a marker-less, single camera ROM assessment, more research is needed. Various established fall risk screening tools exist, and neither Colagiorgio et al.^10^ nor Kargar et al.^11^ compared their ROM assessment to more standard assessments within the United States such as the Stopping Elderly Accidents and Deaths Initiative (STEADI) Checklist, developed by the U.S. Centers for Disease Control and Prevention to screen for fall risk.^12^ Other common fall risk screening tools, including PS path length (which Colagiorio et al.^10^ were unable to report), SPPB scores, and fear of falling via the Short Fall Efficacy Scale International (Short FES-I) must also be included.^13–15^ If clinicians would like to integrate camera-based ROM assessments into their practice as a quick and accessible method of fall risk screening, it is critical to understand how the current clinically established tools relate to ROM assessments. Therefore, the purpose of this study was to assess the relationship between a camera-based lower-limb joint ROM assessment with fall risk as determined by the STEADI checklist, SPPB, PS performance, and Short FES-I among community-dwelling older adults.

## Methods

### Participants

**Figure 1.**
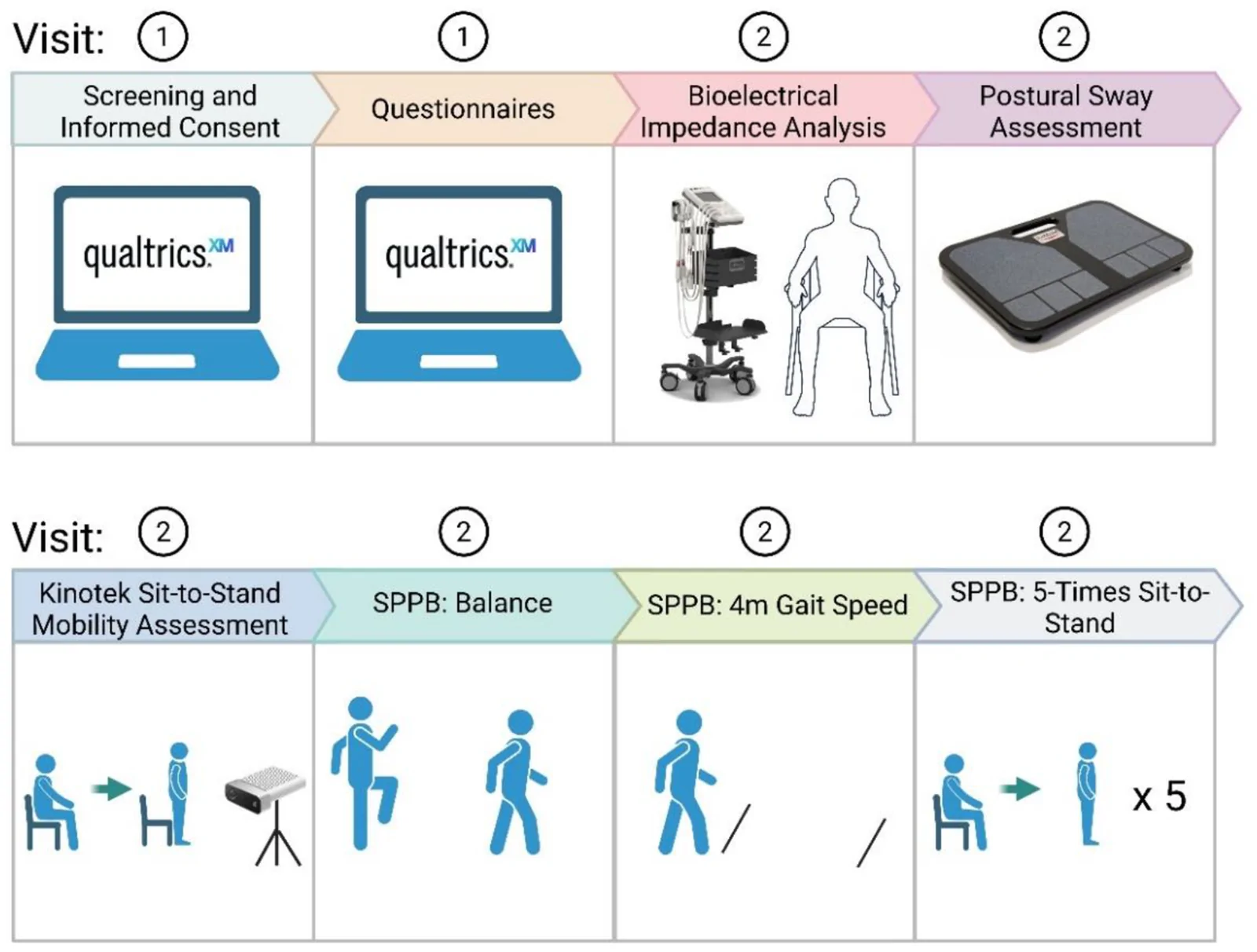
Overview of study procedures. SPPB = Short Physical Performance Battery. Created in BioRender. Lafontant, K. (2026) https://BioRender.com/ul324zf.

We recruited 45 community-dwelling older adults for this cross-sectional study using social media postings, word-of-mouth, and fliers posted in community spaces around the greater Orlando, FL, metropolitan area. All study procedures were conducted between December 2024 and May 2025, approved by the University of Central Florida Institutional Review Board (STUDY00007125), and carried out in accordance with the Declaration of Helsinki. All participants provided written informed consent prior to participation in this study. Participants were included in this study if they were at least 55 years of age, free from implanted pacemakers/defibrillators, not pregnant, and not missing limbs or using prosthetic limbs. This study was divided into an at-home portion and an in-person portion, where questionnaires were completed at home prior to in-person physical assessments (Figure 1).

### At-Home Questionnaires (Visit 1)

Following informed consent, participants completed a demographic questionnaire, the STEADI checklist, and the Short FES-I (in that order) at their own homes using a secure online platform (Qualtrics, Provo, UT, USA). Participants completed these online surveys no more than seven days prior to their in-person study visit. Within the demographic questionnaire, participants self-reported their age, sex, race/ethnicity, history of falls within the last year, and history of fall risk and mobility screenings.

The STEADI checklist is a 12-item questionnaire that assesses physical, emotional, and psychological risk factors related to falls. Within the STEADI algorithm, individuals are classified as high fall risk if they obtain a total score ≥ 4 or if they answer “yes” to at least one of three key questions: “have you fallen in the past year? Do you feel unsteady when standing or walking? Do you worry about falling?”^12^ The STEADI checklist has been validated in diverse populations and is utilized as the primary fall risk screening method by the U.S. Centers for Disease Control and Prevention.^12,16^

The Short FES-I is a validated, 7-item questionnaire designed to assess fall-related self-efficacy, also known as the fear of falling.^17^ Each item focuses on psychological concerns related to falling during several common activities of daily living, such as getting dressed or riding an escalator. Each item is scored from 1 – 4, with a lower score indicating a lower fear of falling for each activity.^17^ The overall score ranges from 7 – 28, with scores ≥ 10 representing a heightened fear of falling.^17^

### In-Person Physical Assessments (Visit 2)

Participants were instructed to arrive at their in-person study visit in comfortable athletic shoes (i.e., sneakers/tennis shoes) and light clothing (e.g., t-shirt, pants, shorts, etc.). Participants were also instructed to arrive having fasted for at least 3 hours, avoided caffeine for at least 12 hours, and abstained from strenuous physical activity/exercise for at least 24 hours. Prior to assessments, participants were encouraged to void their bladder and remove all jewelry. We then measured height and body mass without shoes using a digital physician scale and stadiometer (Health-O-Meter^™^, McCook, IL, USA), from which we calculated body mass index (BMI) as height in meters divided by body mass in kilograms squared.

We then assessed body composition using direct segmental, phase sensitive, multi-frequency bioelectrical impedance analysis (BIA; InBody s10, InBody BWA, Audubon, PA, USA). Participants rested in the seated position for at least 5 minutes prior to each BIA assessment. Participants were seated in a chair with their arms on armrests and their shoes and socks removed. We used an InBody Tissue (InBody BWA, Audubon, PA, USA) to prepare the skin at both middle fingers, thumbs, and ankles immediately inferior to the malleoli. Touch-type electrodes were placed at each of these sites and participants were instructed to remain motionless and silent for the duration of the assessment (~90 seconds). BIA has been demonstrated as a valid and clinically practical estimate of body composition,^18^ and the directly measured bioelectrical variables from the BIA assessment, such as phase angle, have been evidenced to provide non-invasive insight regarding overall cellular health status.^19^ We utilized data from the BIA assessment in this study as additional descriptors for our sample and not as outcome variables.

Following the BIA assessment, participants completed a PS assessment using the Balance Tracking System (BTrackS). The BTrackS consists of the BTrackS Assess Balance software (version 5.5.9) and a portable force plate. The BTrackS measures center-of-pressure postural sway and quantifies the path length following the inverse pendulum model. Participants completed a 20-second familiarization trial, followed by three scored trials, where the data from each of the three trials were averaged together to determine a final score. For each trial, participants stood on the pre-marked force plate with their shoes removed, feet approximately 30cm apart, hands on their hips, and eyes closed. Participants were given approximately 10 seconds of rest between each trial where they were allowed to open their eyes, but their feet remained in the same position during the rest periods. To mitigate the risk of falling, a trained research assistant stood beside each participant during the assessment, and a sturdy piece of furniture was kept within participants’ reach for each trial. The BTrackS as previously been validated against standard force plates for assessing PS among older adults with an ICC_2,1_ of 0.83.^20^

**Figure 2.**
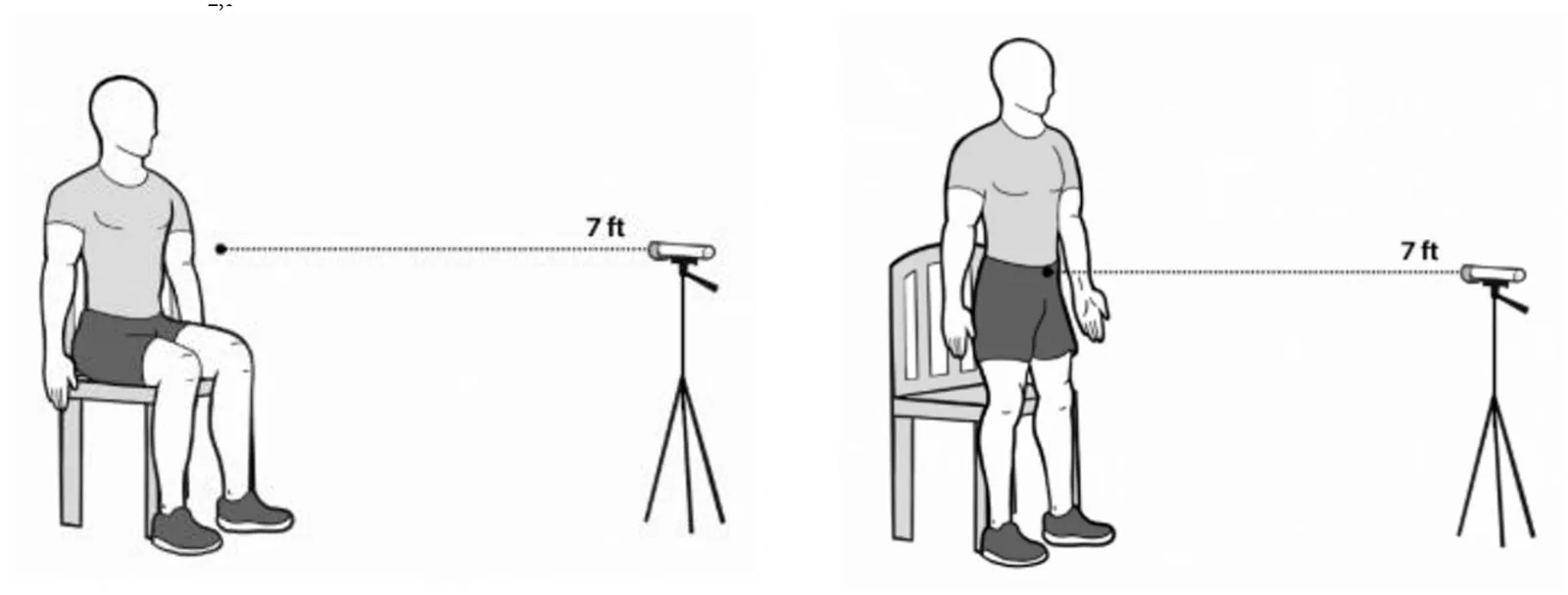
Illustration of the sit-to-stand assessment from the Kinotek software.

After the BTrackS assessment, participants completed a single-camera (Kinect Azure, Model 1880, Microsoft Corporation, Redmond, WA, USA) lower-limb joint ROM assessment via Kinotek software (Kinotek Inc., Portland, ME, USA) during a STS test (Figure 2). Participants were seated in a sturdy chair with no arm rests, their arms at their side with fingers extended towards the ground, and their feet flat on the floor spaced hip-width apart. The chair (seat height = 47cm) was positioned in front of the camera at a 6° angle and 7 feet (~2.13m) away, per Kinotek guidelines. The camera was placed on a tripod set at a level height with the hip (anterior superior iliac spine) for each participant. For the STS, a trained research assistant verbally provided each participant with the standard instructions from Kinotek, instructing participants to start sitting in the chair, facing the camera, stand up, pause for two seconds, then sit back down. Participants began completing STS repetitions once the research assistant verbally said “go,” and participants completed STS repetitions for 10 seconds total to allow the Kinotek to analyze several reps (approximately 2–3 repetitions) of STS prior to the onset of fatigue. The Kinotek uses light detection and ranging (LiDAR) technology and a proprietary artificial intelligence (AI) model to sample movement at a 5 Hz rate, reporting hip flexion, hip adduction, knee flexion, and ankle dorsiflexion averaged for the 10-second duration for each leg, as well as an asymmetry index for each joint movement. The Kinotek software and Microsoft Kinect camera have previously been shown to exhibit strong reliability (ICC_2,k_ = 0.82 – 0.94) in assessing upper- and lower-limb joint ROM during dynamic movements when compared to a standard goniometer;^21,22^. The Microsoft Kinect camera has also been utilized in prior research regarding fall risk assessments.^10,11,23^

Next, participants completed the SPPB, which is a common and validated assessment of physical function for older adults.^13,24^ The SPPB was administered by trained research assistants using a phone application (SPPB Guide, Novartis Pharmaceuticals Corporation, Basel, Switzerland) to standardize instructions, scoring, and timers. The first section of the SPPB is a balance test where participants are asked to maintain three static stances (feet side-by-side, semi-tandem, and tandem) for 10 seconds each. The next section of the SPPB was a 4m normal speed gait assessment. Participants stood behind a marked line on the floor and were instructed to walk past a marked line on the floor 4m directly in front of them at their normal walking speed. The timer began when the research assistant said “go” and ended when at least one foot from the participant crossed the 4m marked line. Participants completed two trials of the gait speed test, with the speed of each trial averaged together for scoring. The final section of the SPPB was a 5-time STS at maximal speed. Participants sat in a sturdy chair with no armrests, their feet flat on the floor, and their hands placed on the opposite shoulder across their chest. Participants were instructed to stand up fully and sit again for a total of five repetitions as quickly as possible. A trained research assistant stood beside the participant to count each correctly completed repetition out loud and ensure that the chair did not move during the test. Participants were instructed to begin once the research assistant said “go” and the test ended once the participants sat in the chair for the fifth time. Each section of the SPPB are scored on a scale of 0 – 4 based on previously published scoring guidelines,^25^ where a lower score indicates poorer physical function. The overall score of the SPPB is determined by adding each section score together, ranging from 0 – 12.^25^ From the SPPB, we also extracted mean gait speed (m/s) from both trials as well as relative muscular power from the 5-time STS, calculated using equation 1 below from Alcazar et al.,^26^ with duration indicating the total duration for the 5-time STS test and units for each variable provided in brackets. Within the present study, the chair height for all participants was 47cm.


eq. 1 $$\text{Relative Mean Power}(W/kg)=\frac{0.9\times 9.81\times (\text{Height}[m]\times 0.5-\text{Chair Height}[m])}{\text{Duration}[s]\times 0.1}$$

### Statistical Analysis

All statistical analyses were conducted using jamovi version 2.5.6.^27,28^ A Shapiro-Wilks test identified non-normally distributed variables, so Spearman’s rank correlation coefficients were used to assess relationships between continuous variables. The STEADI checklist score is quasi-continuous, as a lower score could equate a higher fall risk if that score included a “yes” to one of three key questions, so the STEADI checklist was treated as a categorical variable. A Kruskal-Wallis H test with Dwass-Steel-Critchlow-Fligner (DSCF) post-hoc corrections and epsilon squared (ε^2^) effect sizes were used to compare lower-limb joint ROM between high fall risk and low fall risk groups as determined by the STEADI checklist.^29^ Data are presented as mean ± standard deviation unless otherwise indicated. The threshold for statistical significance was set *a priori* at p < 0.05.

## Results

Table 1 provides demographic participant data. While not a primary or secondary outcome variable, the high fall risk group had a significantly greater body fat percentage than the low fall risk group (p = 0.002, ε^2^ = 0.21).

Table 2 provides the observed ROM for the hip, knee, and ankle joints during the sit-to-stand exercise, as well as data for relative muscular power, PS, gait speed, SPPB, and Short FES-I. While the purpose of this study was not to make comparisons to normative data, it is important to note that the Kinotek software reported normative ranges for hip flexion (90 – 130°), hip adduction (5 – 0°), knee flexion (90 – 140°), and ankle dorsiflexion (15 – 30°).

The high fall risk group according to the STEADI checklist had significantly greater knee flexion asymmetry compared to the low fall risk group (Tables 1 and 3). No other lower-limb joint ROM variables significantly differed between the STEADI checklist fall risk groups. Regarding other fall risk assessments, only fear of falling and lower-limb muscular power differed between groups, with the low fall risk group exhibiting significantly greater lower-limb muscular power and significantly lower fear of falling compared to the high fall risk group (Tables 1 and 3).

Table 4 provides the relationships between lower-limb joint ROM and performance on various fall risk assessments (SPPB, gait speed, relative muscular power, PS, Short FES-I), while Table 5 provides the same relationships after accounting for body fat percentage via an *ad hoc* partial correlation, given the significant difference in body fat percentage between groups. All observed correlation coefficients were small, indicating statistically weak relationships between lower-limb joint ROM and fall risk assessments.

## Discussion

The purpose of this study was to assess the relationship between camera-assessed lower-limb joint ROM and fall risk as determined by the STEADI checklist, SPPB, PS performance, and Short FES-I among community-dwelling older adults. Despite statistical significance for a few outcome variables, such as hip flexion asymmetries being correlated with SPPB and Short FES-I scores, all relationships between all measures of lower-limb joint ROM and all fall risk assessments were relatively small/weak. While accounting for body fat percentage via partial correlations did increase the number of statistically significant relationships between lower-limb joint ROM variables and fall risk assessments, the relationships remained relatively small/weak. Regarding between-group comparisons, only fear of falling (Short FES-I), knee flexion asymmetry, and relative muscular power were significantly different between high and low fall risk groups, with greater fear of falling, greater asymmetries, and lower muscular power among those with higher fall risk (Tables 1 and 2). These results indicate a limited ability to infer fall risk among older adults based on lower-limb joint ROM during the STS, as well as an ability to differentiate those at high and low fall risk via lower-body relative muscular power. Clinicians that are interested in assessing lower-limb joint ROM should do so in tandem with other physical fall risk assessments, such as relative muscular power, while also assessing body fat percentage to include as a covariate.

Previous research utilizing single-camera marker-less systems to assess fall risk largely did not report lower-limb joint ROM, making direct comparisons to prior work difficult.^10,23^ The Microsoft Kinect camera has demonstrated concurrent validity with standard multi-camera 3D motion analysis systems, indicating potential clinical utility as a less cumbersome and inexpensive alternative for clinicians.^32^ Furthermore, previous studies have integrated the Kinect’s ROM data into more complex algorithms with additional insight regarding postural sway and gait mechanics.^10,23^ The present study included gait speed, relative muscular power, and postural sway assessments, demonstrating largely small and weak relationships with lower-limb joint ROM (Table 4) even after accounting for body composition (Table 5). It is possible that lower-limb joint ROM explains a different portion of variance in overall fall risk, leading to improved assessments of fall risk when combined with other measures. However, this study did not test that theory directly, as there is no uniformly accepted criterion measure of fall risk, so whether significant fall risk predictions are observed via regression models would depend largely on the chosen criterion measure, as each test appraises fall risk differently.^14,15^ Even amongst the STEADI checklist, which was developed and promoted by the U.S. Centers for Disease Control and Prevention for clinical use based on previous research,^12,33^ high and low fall risk groups do not appear to significantly differ on common physical function-based fall risk factors, such as gait speed and postural sway (Table 3). Nonetheless, general recommendations for fall risk screening specify a multifactorial approach, incorporating tools that assess different domains of fall risk such as physical, social, and mental risk factors while remaining timely and clinically relevant.^15^ Given the ability for a single-camera system to be used in conjunction with a standard sit-to-stand or gait speed test, this technology may provide clinicians with additional information to factor into their fall risk appraisal.

**Figure 3.**
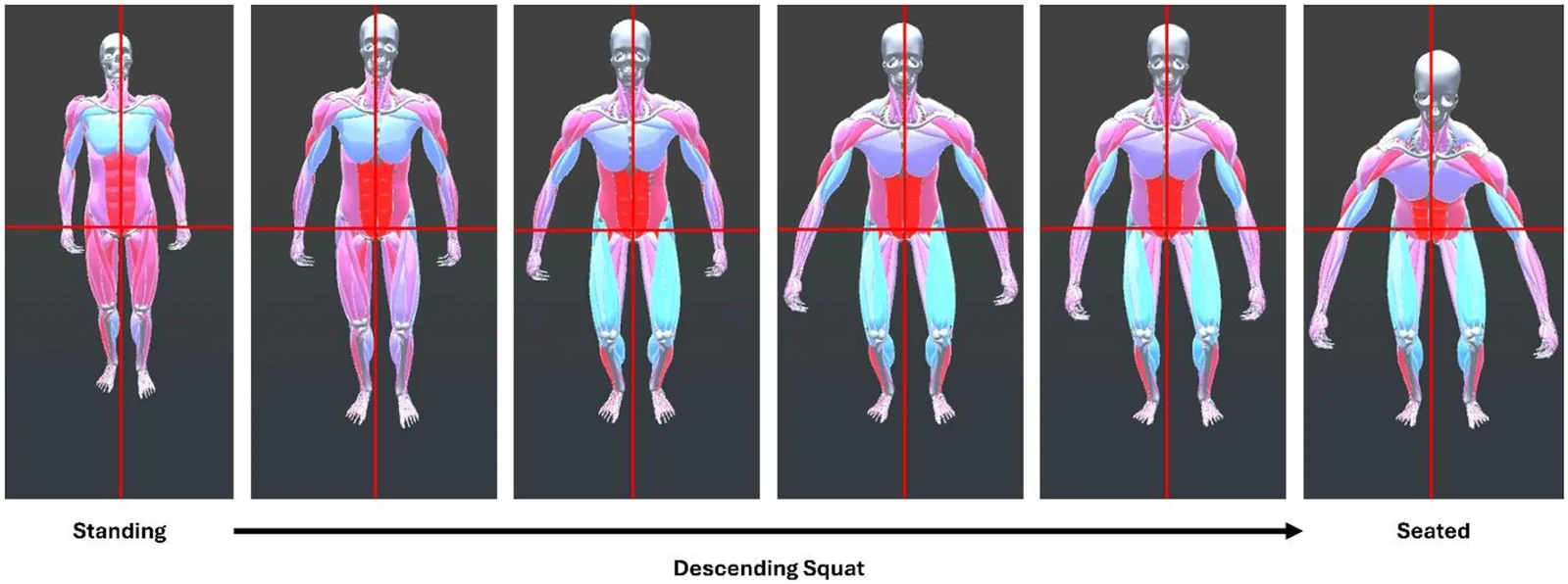
A three-dimensional rendering from Kinotek software of a single participant completing the sit-to-stand exercise. This participant had a hip flexion asymmetry of 8° (greater on the left) and a hip adduction asymmetry of 4° (greater on the right).

Despite the sit-to-stand transition being understood as posing an acute increase in fall risk for older adults,^34^ previous research relating joint kinematics to fall risk has largely done so during walking gait. Wang and Bhatt assessed gait kinematics in middle-aged and older adults, divided into fallers (n = 21) and non-fallers (n = 15) based on fall history within the past year.^35^ Among fallers, they reported smaller trunk and thigh angles and greater kinematic asymmetries, suggesting those as potential risk factors for falling.^35^ Within the present study, ankle dorsiflexion (ϱ = −30, p = 0.048) and hip flexion asymmetry (ϱ = −0.33, p = 0.03) had significant negative relationships with gait speed and SPPB performance, respectively, while hip flexion asymmetry also had a significant positive relationship with Short FES-I scores (i.e., fear of falling; (ϱ = 0.35, p = 0.02). No other joint asymmetries were significantly correlated with the fall risk assessments included in this study, although knee flexion asymmetry was significantly higher in the high fall risk group. The hip flexed/knee flexed/ankle dorsiflexed/forward leaning position is a key step in the sit-to-walk transition as the forward lean aids in propelling the first step in the gait cycle via knee flexion-to-extension (i.e., lower-limb muscular power).^35,36^ An asymmetry in hip and knee flexion during the sit-to-stand movement (Figure 3) may be indicative of a misplaced center of pressure while standing and may contribute to an increased risk of falling when transitioning from standing-to-walking. Further research on this is necessary, as we did not include the standing-to-walking transition in our kinematic assessment. Given the wide variance in ankle dorsiflexion and hip flexion asymmetries, with greater standard deviations than mean value (Table 2), this may be a metric of interest for future research regarding fall risk, as well as lower-limb muscular power given the inherent relationship between lower-limb kinematics and kinetics.

Obesity has been previously evidenced as a relevant factor within fall risk,^37,38^ which was further supported by the present results. Body fat percentage significantly differed between high and low fall risk groups, in-line with prior cross-sectional studies demonstrating a significantly higher fall risk for obese older adults compared to their non-obese counterparts.^37,38^ A recent systematic review and meta-analysis reported 1.78 greater odds for obese older adults with android fat storage (i.e., excessive fat stored in the abdominal region) to experience a fall compared to obese older adults with gynoid fat distribution (i.e., excessive fat stored in the pelvic and upper thigh region), likely due to the greater anterior load from adipose tissue impacting movement patterns.^39^ Furthermore, previous work by Mitchell et al. reported a 30% increased risk of experiencing moderate-to-extreme pain and a 26% likelihood of being physically inactive, both of which may also impact lower-limb joint ROM.^38^ Importantly, we did not test for body fat distribution, pain, or physical activity. However, given body fat percentage functioning as a significant covariate for several comparisons in the present study and the overall body of literature, it may benefit clinicians to incorporate body composition estimates within their fall risk assessments.

While this study emphasized clinical practicality through the inclusion of various fall risk assessments and emerging technology in the single-camera marker-less assessments, our results must be interpreted cautiously with limitations in the study design and execution noted. Our use of the Kinotek system was separate from other assessments such as the gait speed test and SPPB. This was due to the Kinotek software using a set timing system for the assessment that would not have aligned with these standardized tests, as well as the need for a research assistant to spot participants for safety during the SPPB and gait speed tests, potentially influencing camera-based measures. Nonetheless, future research should aim to utilize camera-based assessments in tandem with other assessments, employing harness or handrail systems to still address participant safety. The Kinotek software’s AI model is also proprietary, potentially limiting reproducibility of our results with other software. Furthermore, given the small effect sizes for lower-limb joint ROM, we likely lacked statistical power for several comparisons, so our results should be interpreted cautiously and more so as exploratory. Our sample was also largely White and female, so generalizability to other races/ethnicities and men must be done cautiously. Previous research has noted an unexplained difference in fall risk among American older adults of different races,^40,41^ with more research needed to better understand the mechanisms underlying these trends. Additionally, we chose to use the STEADI checklist to subdivide our sample into high and low fall risk given its endorsement and use by the United States’ Centers for Disease Control and Prevention in clinical practice guidelines.^8,12^ However, previous work from our research group suggests that the STEADI checklist may categorize individuals as high fall risk to a greater extent than other fall risk assessments,^15^ so it is likely that our results would change if a different assessment was used to categorize high and low fall risk. Lastly, the absence of sex-based comparisons was a delimitation, as none of the utilized fall risk assessments differentiate between sexes when determining fall risk, as any potential differences between male and female participants would likely not have any applicability in clinical settings during fall risk screenings.

## Conclusions

Lower-limb joint ROM as measured by single-camera marker-less systems during a sit-to-stand may not be a strong indicator of fall risk among older adults but may be clinically useful if combined with other fall risk measures, such as lower-body relative muscular power. While we demonstrated significantly lower muscular power and significantly higher hip and knee asymmetries among those with high fall risk, further research is needed with larger and more diverse samples to investigate the potential for hip flexion, knee flexion, and ankle dorsiflexion asymmetries to serve as indicators of fall risk in the standing-to-walking transition. If clinicians can utilize camera systems to assess lower-limb joint ROM, it may be more practical to do so during other routine fall risk/physical function assessments to maximize the amount of clinically relevant information gained.

## Figures and Tables

**Table 1.** Participant characteristics (N = 45).

	All (N = 45)	High STEADI Fall Risk (n = 24)	Low STEADI Fall Risk (n = 21)
Age (years)	76.5 ± 6.1	76.8 ± 6.3	76.3 ± 5.9
	(63 – 94)	(63 – 87)	(70 – 94)
Weight (kg)	71.3 ± 14.8	70.2 ± 15.3	72.5 ± 14.4
	(45.4 – 106.3)	(45.4 – 106.3)	(49.1 – 100.8)
BMI (kg/m^2^)	26.4 ± 4.0	27.1 ± 4.7	25.6 ± 3.1
	(17.2 – 38.2)	(17.2 – 38.2)	(19.8 – 29.6)
Body Fat Percentage (%)	34.7 ± 8.1	38.1 ± 8.1	30.8 ± 6.3
	(18.3 – 52.0)	(18.3 – 52.0)	(21.9 – 42.9)
Phase Angle (°)	4.6 ± 0.7	4.5 ± 0.6	4.7 ± 0.8
	(3.3 – 6.4)	(3.3 – 5.9)	(3.5 – 6.4)
Sex	Male: 13 (28.9%)	Male: 3 (12.5%)	Male: 10 (47.6%)
	Female: 32 (71.1%)	Female: 21 (87.5%)	Female: 11 (52.4%)
Race/Ethnicity	A: 1 (2.2%)	A: 0 (0%)	A: 1 (4.8%)
	AA: 2 (4.4%)	AA: 2 (8.3%)	AA: 0 (0%)
	H: 1 (2.2%)	H: 1 (4.2%)	H: 0 (0%)
	NHW: 41 (91.2%)	NHW: 21 (87.5%)	NHW: 20 (95.2%)
Falls in previous year	None: 27	None: 7 (29.2%)	None: 21 (100%)
	Once: 6	Once: 6 (25%)	Once: 0 (0%)
	Twice: 8	Twice: 8 (33.3%)	Twice: 0 (0%)
	Three or more: 3	Three or more: 3 (12.5%)	Three or more: 0 (0%)
Has a medical professional ever assessed your balance?	Yes: 19	Yes: 11 (45.8%)	Yes: 8 (38.1%)
	No: 21	No: 9 (37.5%)	No: 12 (57.1%)
	Not sure: 5	Not sure: 4 (16.7%)	Not sure: 1 (4.8%)
Has a medical professional ever assessed your mobility?	Yes: 11	Yes: 8 (33.3%)	Yes: 3 (14.3%)
	No: 27	No: 12 (50%)	No: 15 (71.4%)
	Not sure: 7	Not sure: 4 (16.7%)	Not sure: 3 (14.3%)

Note. A = Asian; AA = African American; H = Hispanic; NHW = Non-Hispanic White; BMI = Body Mass Index. Data are presented as mean ± standard deviation, (range), or n (%).

**Table 2.** Lower-limb joint range-of-motion characteristics.

Variable	All (N = 45)	High STEADI Fall Risk (n = 24)	Low STEADI Fall Risk (n = 21)
Left Hip Flexion (°)	76.2 ± 10.9	76.4 ± 11.9	76.0 ± 9.9
	(46 – 105)	(48 – 105)	(46 – 95)
Right Hip Flexion (°)	76.7 ± 11.2	77.3 ± 12.6	75.9 ± 9.7
	(46 – 109)	(48 – 109)	(46 – 90)
Hip Flexion Asymmetry (°)	2.56 ± 2.70	3.2 ± 3.2	1.8 ± 1.9
	(0 – 13)	(0 – 13)	(0 – 7)
Left Hip Adduction (°)	8.87 ± 6.36	9.6 ± 7.2	8.0 ± 5.3
	(0 – 29)	(0 – 29)	(0 – 22)
Right Hip Adduction (°)	6.60 ± 4.55	6.6 ± 4.3	6.6 ± 4.9
	(0 – 22)	(1 – 22)	(0 – 19)
Hip Adduction Asymmetry (°)	4.87 ± 4.16	4.6 ± 4.3	5.1 ± 4.0
	(0 – 18)	(0 – 17)	(0 – 18)
Left Knee Flexion (°)	94.1 ± 12.3	93.5 ± 12.8	94.8 ± 12.1
	(59 – 115)	(62 – 115)	(59 – 112)
Right Knee Flexion (°)	93.6 ± 12.0	92.8 ± 11.6	94.6 ± 12.7
	(54 – 117)	(72 – 117)	(54 – 109)
Knee Flexion Asymmetry (°)	3.76 ± 2.59	4.5 ± 2.5	2.9 ± 2.4
	(0 – 10)	(1 – 10)	(0 – 9)
Left Ankle Dorsiflexion (°)	21.5 ± 8.36	20.9 ± 9.6	22.1 ± 6.9
	(2 – 39)	(2 – 39)	(11 – 36)
Right Ankle Dorsiflexion (°)	22.2 ± 7.97	21.2 ± 8.3	23.3 ± 7.6
	(8 – 43)	(8 – 43)	(11 – 38)
Ankle Dorsiflexion Asymmetry (°)	3.58 ± 3.83	3.3 ± 2.4	3.9 ± 5.1
	(0 – 24)	(0 – 10)	(0 – 24)
Muscular Power (W/kg)	3.2 ± 0.9	2.8 ± 0.6	3.6 ± 1.0
	(1.9 – 6.0)	(1.9 – 4.0)	(2.1 – 6.0)
PS (cm)	32.0 ± 15.3	31.4 ± 16.4	32.6 ± 14.3
	(15 – 71)	(15 – 71)	(16 – 61)
Gait Speed (m/s)	0.771 ± 0.143	0.748 ± 0.151	0.798 ± 0.131
	(0.468 – 1.090)	(0.468 – 1.090)	(0.592 – 1.059)
SPPB	10.5 ± 1.7	10.1 ± 1.9	11.0 ± 1.2
	(5 – 12)	(5 – 12)	(7 – 12)
Short FES-I	10.3 ± 4.7	11.9 ± 5.1	8.5 ± 1.5
	(7 – 23)	(7 – 23)	(7 – 12)

Note. PS = postural sway path length; SPPB = Short Physical Performance Battery; Short FES-I = Short Fall Efficacy Scale International.

**Table 3.** Comparison of lower-limb joint range-of-motion and fall risk assessment scores between STEADI checklist high and low risk older adults (N = 45).

	Left		Right		Asymmetry	
Lower-Limb ROM	p	ε^2^	p	ε^2^	p	ε^2^
Hip Flexion	0.784	0.002	0.802	0.001	0.086	0.067
Hip Adduction	0.732	0.003	0.740	0.003	0.430	0.014
Knee Flexion	0.640	0.005	0.327	0.022	0.021	0.121
Ankle Dorsiflexion	0.616	0.006	0.311	0.023	0.695	0.004
Fall Risk Assessments	p	ε^2^				
SPPB	0.078	0.071				
Gait Speed	0.215	0.035				
Muscular Power	0.004	0.187				
Postural Sway	0.724	0.003				
Short FES-I	0.010	0.152				

Note. ROM = range-of-motion; STEADI = Stopping Elderly Accidents, Deaths, and Injuries; SPPB = Short Physical Performance Battery; Short FES-I = Short Fall Efficacy Scale International. Effect sizes are given as epsilon squared (ε^2^) where 0.02, 0.15, and 0.35 represent small, medium, and large effect sizes, respectively^30,31^.

**Table 4.** Relationship between lower-limb joint range-of-motion and fall risk assessments (N = 45).

	Left		Right		Asymmetry	
SPPB	ϱ	p	ϱ	p	ϱ	p
Hip Flexion	−0.12	0.44	−0.26	0.09	−0.33	0.03
Hip Adduction	0.22	0.16	0.16	0.28	0.08	0.59
Knee Flexion	0.14	0.37	−0.21	0.16	0.01	0.96
Ankle Dorsiflexion	0.28	0.06	−0.21	0.16	−0.24	0.12
Gait Speed						
Hip Flexion	−0.25	0.10	−0.21	0.17	−0.25	0.10
Hip Adduction	0.14	0.37	0.23	0.14	0.08	0.59
Knee Flexion	−0.31	0.04	−0.22	0.15	0.02	0.91
Ankle Dorsiflexion	−0.16	0.29	−0.28	0.06	−0.30	0.048
Muscular Power						
Hip Flexion	−0.10	0.52	−0.09	0.54	−0.26	0.08
Hip Adduction	0.14	0.36	0.09	0.57	−0.03	0.82
Knee Flexion	0.10	0.50	0.16	0.30	−0.07	0.65
Ankle Dorsiflexion	0.18	0.24	0.16	0.28	−0.09	0.54
PS						
Hip Flexion	0.25	0.09	0.23	0.12	0.14	0.35
Hip Adduction	−0.29	0.05	−0.38	0.01	−0.24	0.12
Knee Flexion	0.03	0.86	0.16	0.29	0.08	0.59
Ankle Dorsiflexion	−0.28	0.07	−0.13	0.39	0.25	0.10
Short FES-I						
Hip Flexion	−0.12	0.44	−0.14	0.35	0.35	0.02
Hip Adduction	0.22	0.16	−0.05	0.74	0.01	0.97
Knee Flexion	0.14	0.36	−0.004	0.98	0.24	0.11
Ankle Dorsiflexion	0.28	0.06	0.15	0.32	−0.18	0.25

Note. PS = postural sway path length, Short FES-I = Short Fall Efficacy Scale International.

**Table 5.** Relationship between lower-limb joint range-of-motion and fall risk assessments accounting for body fat percentage (N = 45).

	Left		Right		Asymmetry	
SPPB	ϱ	p	ϱ	p	ϱ	p
Hip Flexion	−0.37	0.01	−0.35	0.02	−0.33	0.03
Hip Adduction	0.003	0.98	0.24	0.12	−0.04	0.81
Knee Flexion	−0.32	0.03	−0.31	0.04	0.04	0.80
Ankle Dorsiflexion	−0.15	0.34	−0.25	0.10	−0.25	0.11
Gait Speed						
Hip Flexion	−0.32	0.04	−0.30	0.05	−0.24	0.12
Hip Adduction	0.05	0.76	0.31	0.04	−0.04	0.79
Knee Flexion	−0.39	0.01	−0.32	0.03	0.05	0.74
Ankle Dorsiflexion	−0.18	0.24	−0.33	0.03	−0.31	0.04
Muscular Power						
Hip Flexion	−0.17	0.28	−0.19	0.23	−0.26	0.09
Hip Adduction	0.04	0.82	0.17	0.26	−0.21	0.18
Knee Flexion	0.06	0.70	0.08	0.60	−0.04	0.80
Ankle Dorsiflexion	0.20	0.19	0.16	0.31	−0.09	0.56
PS						
Hip Flexion	0.25	0.10	0.23	0.13	0.14	0.35
Hip Adduction	−0.30	0.045	−0.38	0.01	−0.25	0.10
Knee Flexion	0.03	0.87	0.16	0.29	0.08	0.59
Ankle Dorsiflexion	−0.28	0.07	−0.13	0.40	0.25	0.11
Short FES-I						
Hip Flexion	−0.09	0.57	−0.10	0.52	0.34	0.02
Hip Adduction	0.33	0.03	−0.11	0.49	0.12	0.43
Knee Flexion	0.19	0.22	0.06	0.68	0.23	0.14
Ankle Dorsiflexion	0.30	0.049	0.18	0.23	−0.20	0.20

Note. PS = postural sway path length, Short FES-I = Short Fall Efficacy Scale International. Body fat percentage was included as a control variable within a partial correlation.
